# An Orbicularis Oculi-Related Band as a Potential Modifier of Tear-Trough Visibility: A Hypothesis-Generating Observation of a Layered Boundary in a Patient †

**DOI:** 10.3390/jcm15166268

**Published:** 2026-08-13

**Authors:** Jurand Domański

**Affiliations:** Department of Preclinical Sciences, Pharmacology and Medical Diagnostics, Faculty of Medicine, Wrocław University of Science and Technology, ul. Hoene-Wrońskiego 13c, 50-376 Wrocław, Poland; jurand.dom@gmail.com

**Keywords:** tear trough, orbicularis oculi, orbicularis oculi-related band, high-frequency ultrasound, color Doppler, multimodal imaging, clinical anatomy, nasomaxillary anatomy

## Abstract

**Background/Objectives:** A surface feature first noticed after aesthetic treatment may be misclassified as treatment-created. This single-patient observation examined a stable medial tear-trough contour noticed after midface volumization. The aims were to map its clinically continuous course across anatomical layers and assess whether a superficial orbicularis oculi (OOc)-related component could modify its visibility. **Methods**: Clinical examination, palpation-guided surface marking, retrospective photography, cone-beam computed tomography (CBCT), thin-section computed tomography (CT), high-frequency ultrasound, and qualitative color Doppler were correlated using the marked course as a common spatial reference. **Results**: Diffuse fullness decreased after clinically indicated hyaluronidase treatment, whereas a narrower palpable line persisted; retrospective photographs showed similar earlier topography. CT/CBCT identified a bilateral asymmetric osseous correlate confined to the superior medial segment. Ultrasound identified a superficial band-like structure along the medial and inferior border of OOc with sonographic features compatible with an OOc-related muscular component; it was more superficial on the left and separated from the levator labii superioris alaeque nasi/levator labii superioris (LLSAN/LLS) field by adipose tissue. Qualitative color Doppler localized a deeper angular venous pathway and a superficial tributary passing beneath the band. The clinical course was continuous, but its correlates changed by segment and plane. **Conclusions**: In this patient, the contour was best understood as a layered boundary rather than a single substrate. Changes in surrounding volume may have increased its visibility, while the OOc-related band may have sharpened the edge adjacent to the tear-trough hollow. Layer-specific classification may be useful before corrective treatment is considered.

## 1. Introduction

In aesthetic medicine, a new surface finding after treatment is usually interpreted through two legitimate frames: normal population anatomy and the expected effects or complications of a recent procedure. Both are necessary for planning treatment and recognizing adverse outcomes [1,2,3], but neither establishes origin when a patient-specific anatomical configuration becomes visible, palpable, or aesthetically unfavorable only under particular in vivo conditions. After midface volumization, chronology can therefore make retained material, tissue reaction, or another procedural change seem more likely before the topography and contributing layer are understood. Treatment can alter local tissue conditions and clinical attention, making a pre-existing configuration newly legible without making it newly formed.

The medial lower eyelid–cheek transition is particularly susceptible to this problem because limited soft-tissue buffering allows small differences between adjacent planes to produce conspicuous surface effects [3,4,5]. A subtle configuration may remain clinically quiet under one set of conditions and become distinct under another when osseous microrelief, superficial orbicularis oculi (OOc)-related support, fat-plane transitions, vascular relationships, and mimetic continuity converge along the same surface course. Once visible, a defined edge can increase contrast with the neighboring hollow and make the tear trough appear deeper even without additional volume loss.

The relevant anatomy has largely been described in separate frameworks: tear-trough and lid–cheek anatomy, retaining and fibrous support, facial fat compartments, OOc architecture, nasomaxillary morphology, and angular vessels [6,7,8,9,10,11]. The unresolved problem is their clinically anchored integration in vivo. Descriptions of the individual structures do not explain how one clinically continuous contour can arise from correlates that change by segment and plane. The question is therefore not simply which structure is present, but how the layers relate along the same visible course.

This hypothesis-generating anatomical–clinical observation used a visible and palpable medial infraorbital–maxillary course as a common spatial reference for correlating surface anatomy with living-tissue architecture. Clinical examination and palpation-guided marking, retrospective photography, cone-beam computed tomography (CBCT), thin-section computed tomography (CT), high-frequency ultrasound, qualitative color Doppler, and anatomical literature were integrated to map the course across layers, define the explanatory range of each modality, and assess the possible contribution of a superficial OOc-related component to tear-trough visibility.

## 2. Materials and Methods

### 2.1. Initial Observation and Analytical Frame

This multimodal anatomical–clinical observation in a single patient examined a reproducible medial infraorbital–maxillary surface course that was visible, palpable, and suitable for skin marking. The finding was assessed after previous deep hyaluronic acid (HA) volumization of the midface over the anterior maxillary body and adjacent infraorbital–maxillary region. For clarity, marked surface course denotes the clinically visible and palpable contour used as the spatial reference; OOc-related muscular component denotes the superficial structure interpreted as muscular after concordant sonographic morphology and topography; OOc-related band is retained as a concise topographic descriptor when comparison with the anatomical literature is intended.

Treatment history framed the initial differential diagnosis. The analysis proceeded from clinical definition and surface marking through retrospective photographic review and anatomical and anthropological consultation to assessment of possible osseous, soft-tissue, and vascular correlates across successive modalities. The unit of analysis was the clinically defined course rather than the entire infraorbital region. The analysis was descriptive and did not include inferential statistics.

**Figure 1:**
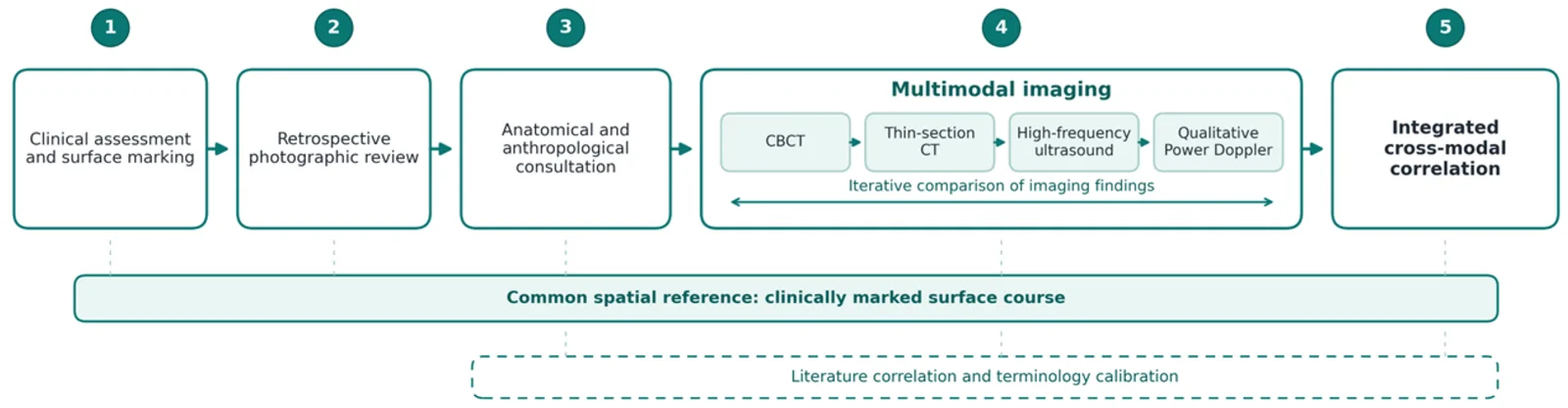
Analytical workflow of the multimodal anatomical–clinical assessment. The diagram summarizes the sequence from clinical assessment and surface marking through retrospective photographic review, anatomical and anthropological consultation, multimodal imaging with cone-beam computed tomography (CBCT), thin-section computed tomography (CT), high-frequency ultrasound, and qualitative color Doppler, iterative comparison of imaging findings, and integrated cross-modal correlation. The clinically marked surface course served as the common spatial reference across modalities. Literature correlation and terminology calibration supported interpretation during the later analytical stages.

Available records established the following relative clinical sequence: pre-treatment photographs; midface HA volumization; recognition of the contour as a distinct concern; and clinically indicated hyaluronidase followed by two further targeted treatments at two-week intervals. Multimodal imaging was performed approximately one year after completion of the hyaluronidase-treatment sequence. The subsequent analytical sequence is summarized in Figure 1.

### 2.2. Surface Marking and Clinical Documentation

Inspection and palpation assessed the visible course, its detectability on palpation, its relationship to diffuse tissue fullness, and its relationship to the medial canthus, anterior lacrimal crest, frontal process of the maxilla, medial inferior orbital rim, anterior maxillary surface, and midpupillary line.

The surface course was marked and photographed before imaging comparison. The marked course then served as the common spatial reference for cross-modal comparison.

### 2.3. Retrospective Photographic Assessment

Available pre-treatment photographs were reviewed to distinguish the time of clinical recognition from the earlier presence of a similar contour. The reviewed material included patient-provided photographs and images retained in the clinical documentation. Older photographs dating to approximately six years before treatment were reviewed only for the presence of the contour; a more detailed assessment used serial photographs from the preceding year and an image obtained immediately before the procedure. Presence, conspicuity, continuity, course, and relationship to the medial tear trough and lower eyelid–cheek transition were assessed.

Available photographs obtained before and approximately 10–14 days after the earlier symmetrical botulinum neurotoxin (BoNT) treatment of the bunny-line region were compared using the most closely matched available views for facial position and expression. The comparison assessed side-to-side differences in the nasolabial fold, oral commissure, and medial midface response. Differences in soft-tissue volume, body weight, facial expression, and previous treatment in adjacent regions were treated as contextual influences on visibility. A later clinical reassessment was also reviewed qualitatively.

### 2.4. Anatomical and Anthropological Consultation and Orientation of Subsequent Analysis

Photographic documentation, palpation findings, preliminary imaging observations, and the topographic course of the finding were reviewed during anatomical and anthropological consultations.

The consultations considered whether the course and palpatory features could reflect an osseous, soft-tissue, or combined configuration in the nasomaxillary and medial infraorbital regions. Their role was to refine subsequent imaging questions and the literature search without assigning tissue identity or validating a predefined diagnosis.

### 2.5. CBCT and Thin-Section CT Assessment

CBCT and thin-section CT were used to assess a possible osseous correlate of the marked course. The course was related to regional landmarks, and three-dimensional reconstructions and axial sections were analyzed to determine whether corresponding osseous morphology was present along its full extent or only within a segment.

CBCT was performed first to assess three-dimensional maxillofacial osseous morphology. Because it did not permit adequate bilateral assessment or provide sufficient detail to depict subtle nasomaxillary surface relief, clinically indicated thin-section CT was subsequently obtained for more precise bilateral evaluation, axial assessment of the bony surface, and limited soft-tissue correlation. Both examinations formed part of the clinical diagnostic evaluation and were not acquired solely for research or publication.

Named anatomical structures provided regional orientation; morphology was assessed without prior assignment to a specific osseous variant. Acquisition, reconstruction, device, software, measurement, and spatial-resolution details are reported in the Supplementary Technical Note.

### 2.6. High-Frequency Ultrasound and Qualitative Color Doppler

High-frequency ultrasound was used to identify a soft-tissue correlate of the visible and palpable course and define its relationship with adjacent anatomical layers. The examination used a linear high-frequency transducer and a superficial dermatologic preset adapted to facial soft-tissue imaging. Regional orientation and recognized facial structures were cross-referenced against the ultrasonographic atlas of Kim et al. [12] and established facial-ultrasound guidance [13]. The probe was then aligned with the marked course in long- and short-axis orientations.

B-mode ultrasound assessed the skin, adipose tissue, mimetic muscles, vessels, and visible bony surface features. A candidate muscular correlate was compared with adjacent mimetic muscles. Muscular interpretation was based on a concordant set of features: echogenicity comparable with adjacent facial muscles; an organized directional fibrillar pattern rather than homogeneous echotexture; reproducibility in long- and short-axis views; continuity within the examined segments; and consistent relationships to OOc, the levator labii superioris alaeque nasi/levator labii superioris (LLSAN/LLS) field, intervening adipose tissue, and adjacent vessels. Repeated short probe placements maintained spatial correspondence between the clinical marking and the sonographic planes.

Qualitative color Doppler was applied in the same areas to localize vascular signals relative to the clinical course and the layers visible on B-mode imaging. The assessment determined whether a vascular signal coincided with the marked line or occupied an adjacent or deeper plane. Device, probe, and image-specific acquisition parameters are reported in the Supplementary Technical Note.

All clinical marking, ultrasound, image review, measurements, and cross-modal interpretation were performed by the author; CBCT and CT were acquired by technologists using parameters jointly established with the author.

### 2.7. Anatomical Reference Fields and Literature Correlation

Literature selection was iterative and followed the clinical assessment, anatomical consultation, and imaging findings. It included lower-eyelid and tear-trough anatomy, the lid–cheek junction, nasomaxillary morphology, OOc-related support and fascicular anatomy, fat planes, mimetic continuity, and the angular vascular pathway.

The literature was used to define anatomical plausibility, terminology, and interpretive limits. HA filler and facial-ultrasound sources contextualized the initial differential diagnosis and imaging strategy, whereas interpretation remained grounded in the multimodal observations in this patient. Published anatomical labels were treated as reference fields rather than as evidence of patient-specific structural identity.

## 3. Results

### 3.1. Clinical Observation and Surface Effect

**Figure 2:**
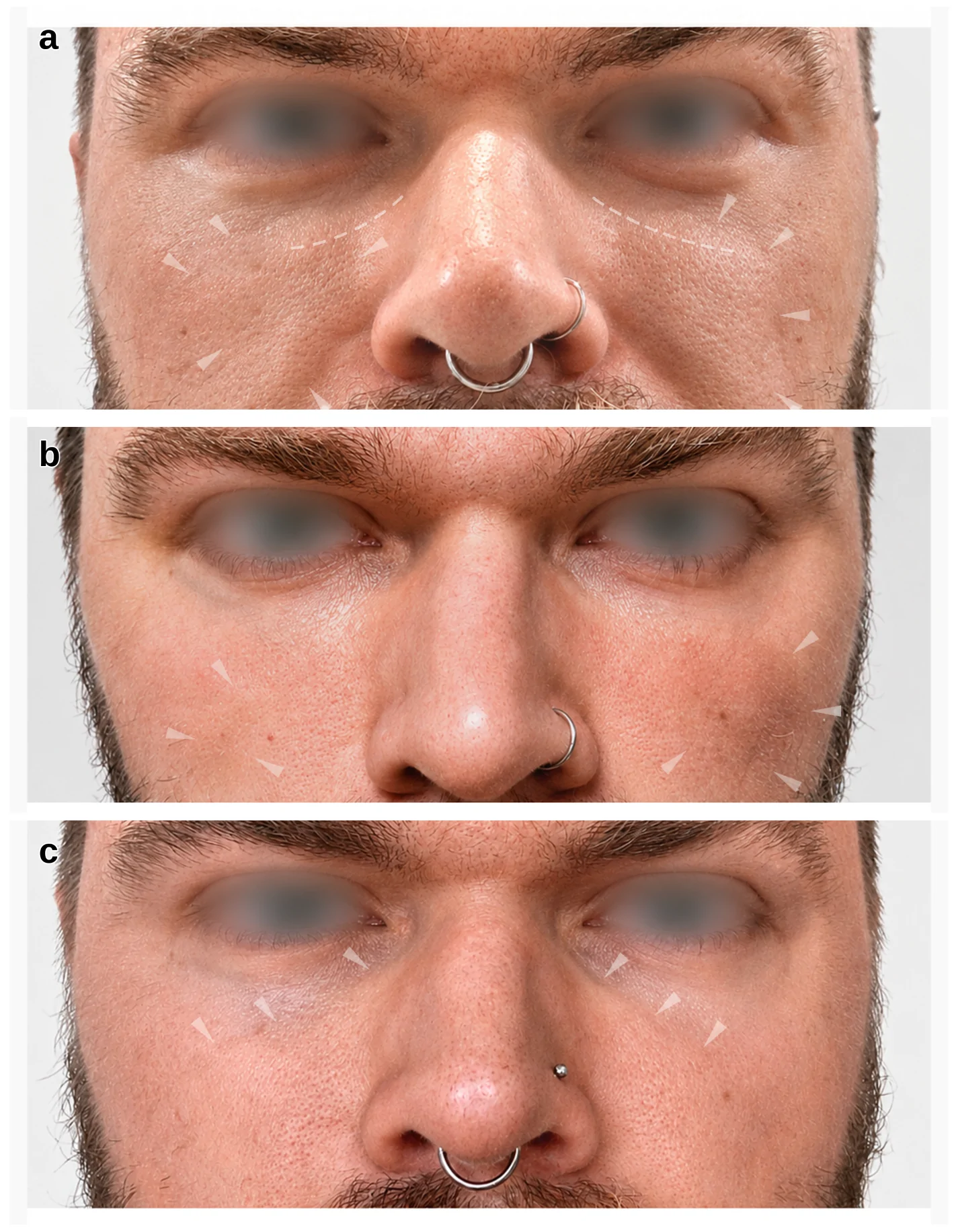
Clinical dissociation between the variable volume-related component and the stable linear contour. (**a**) Appearance before hyaluronidase treatment. Arrows indicate diffuse cushion-like infraorbital fullness; the line marks the firm palpable course. (**b**) Appearance after hyaluronidase treatment. Diffuse fullness decreased, whereas the palpable line remained (arrowheads). Bruising within the treatment area is shown as a procedural safety observation and may reflect injury to a superficial vessel. (**c**) Retrospective photograph showing a similar contour (arrowheads) extending from the medial tear-trough region beyond the midpupillary line.

In this 32-year-old patient, a reproducible medial infraorbital–maxillary line was visible and palpable and was more sharply demarcated on the left. Clinical assessment distinguished a variable, diffuse, cushion-like fullness from a narrower, stable linear finding (Figure 2a,b). The course initially indicated by the patient was confirmed on clinical examination by two physicians. On the left, an approximately 2 mm edge began near the medial canthus and anterior lacrimal crest and descended obliquely through the medial tear-trough region toward the middle-lower infraorbital–maxillary segment.

The surface appearance differed by segment. Superiorly, the line formed the anterior clinical edge of the medial tear trough, with the hollow visible between the edge, lacrimal margin, and medial inferior orbital rim. More inferiorly, it continued as a separate line-like change in infraorbital–maxillary skin topography.

After clinically indicated hyaluronidase treatment, the diffuse fullness decreased, whereas the linear finding remained palpable. Two further targeted treatments at two-week intervals did not eliminate its palpability. The diffuse fullness and narrower line therefore showed different clinical behavior: the former decreased, whereas the latter remained stable and palpable.

### 3.2. Retrospective Presence and Modulation of Clinical Readability

Available retrospective photographs showed a similar bilateral contour before the post-treatment episode (Figure 2c), involving the anterior medial tear-trough edge and the more inferior infraorbital–maxillary segment. The contour had not previously been consciously classified by the patient as a separate aesthetic concern.

After treatment, the clinical readability of selected segments increased, whereas the anterior medial tear-trough edge remained broadly similar. Contour conspicuity also varied with tissue conditions and facial expression. At assessment 10–14 days after symmetrical BoNT treatment of the bunny-line region, the left side showed a smaller clinical response than the right: the left nasolabial fold flattened less, and the left oral commissure remained relatively higher. The same direction of side-to-side difference was again observed at the later clinical reassessment.

### 3.3. CT/CBCT: Superior Osseous Correlate and Soft-Tissue Continuation

**Figure 3:**
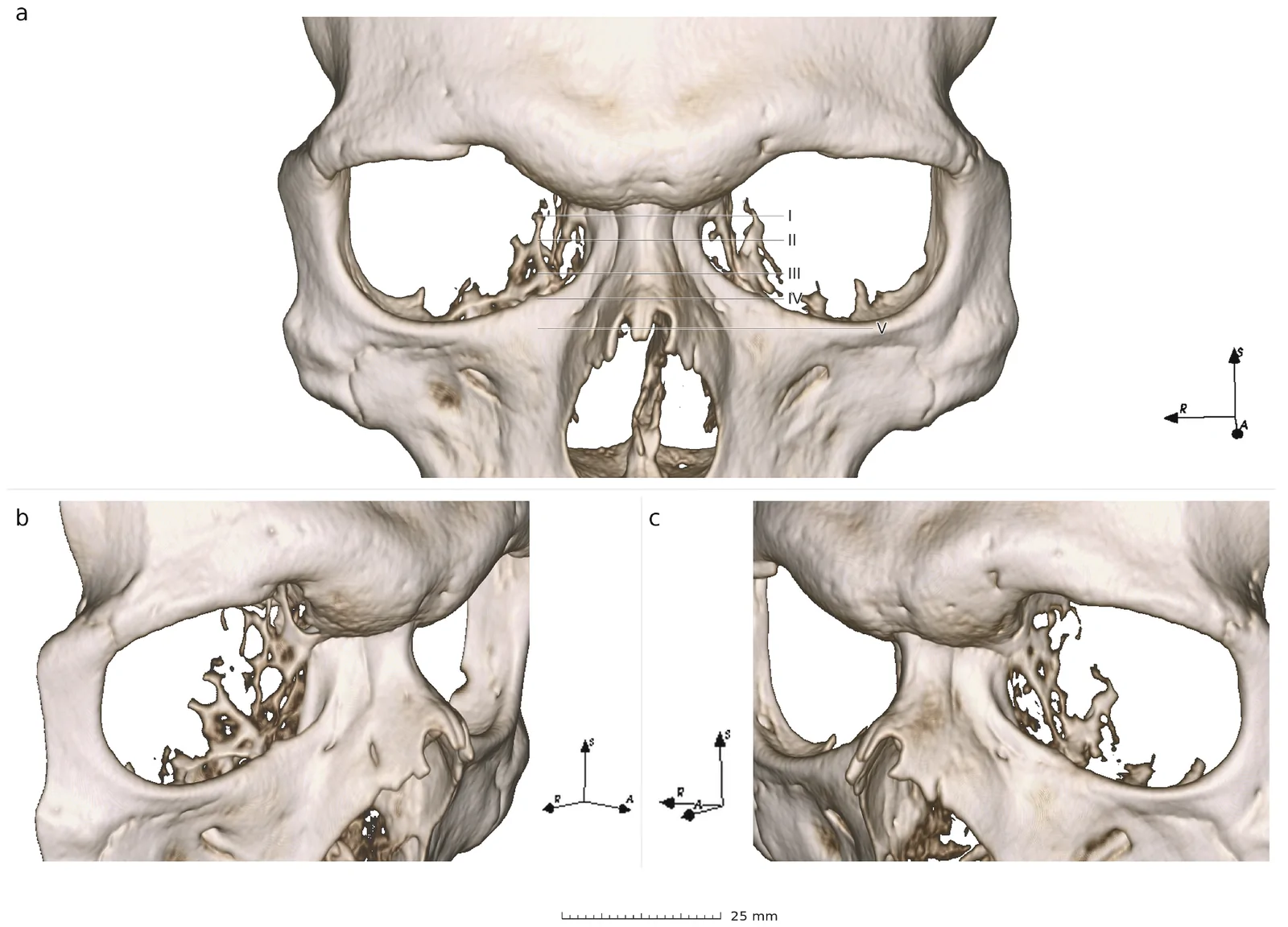
Three-dimensional reconstruction of the nasomaxillary region and surface morphology. (**a**) Anterior view. Lines I–V mark the reference levels corresponding to panels (a–e) in Figure 4. The superior medial orbital–lacrimal region shows a steep bony wall with a sharp edge, small bony projections, and ledge-like elevations. (**b**) Right oblique view. A narrower gutter-like depression is bordered anteriorly by a sharp ledge-like bony elevation; the more pronounced angulation between the lateral surface of the frontal process of the maxilla (FPM) and the anterior maxillary body persists inferiorly, producing further continuation of the depression. (**c**) Left oblique view. The depression is wider and flatter, with a less prominent anterior boundary; the FPM–maxillary body angulation is less pronounced and the depression progressively widens, flattens, and fades inferiorly. In both oblique views, small foramina and fine surface lines are visible (probably impressions or traces of subperiosteal vessels, morphologically reminiscent of Macalister’s “vascular line” [14]). The oblique views demonstrate asymmetric bony-plane angulation and a different inferior course of the depressions.

**Figure 4:**
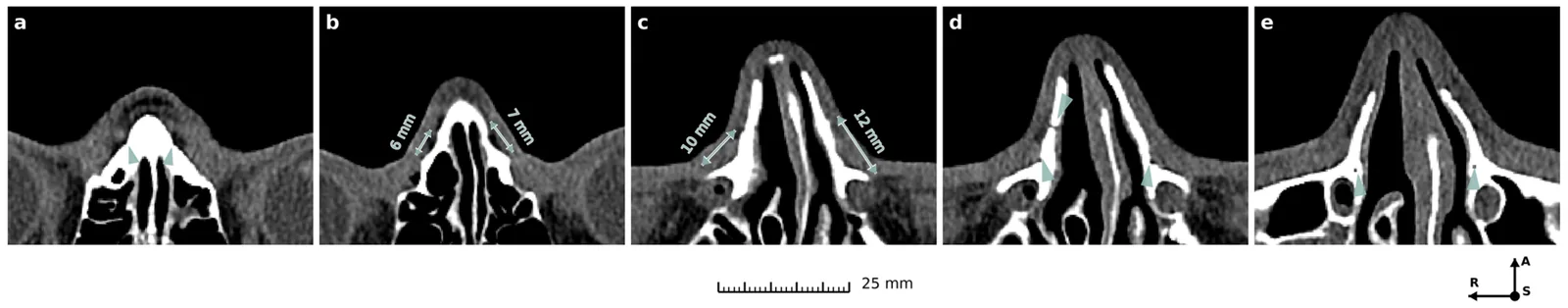
Axial CT sections of the nasomaxillary region at reference levels I–V marked in Figure 3a. (**a**) Level I: Arrowheads indicate the superior onset of bilateral bony depressions within the superior nasomaxillary field. (**b**) Level II: At a lower level, the right depression has a sharply demarcated V-shaped profile and measures approximately 0.7 mm in depth; the left is wider, less steeply demarcated, and approximately 1.7 mm deep. (**c**) Level III: At the level of greatest expression, the right depression measures approximately 1.9 mm and is bordered by a sharp, narrow anterior bony elevation projecting up to approximately 1.0 mm above the lateral FPM surface; the left depression is broader and flatter, measures approximately 2.0 mm, and has a corresponding elevation of approximately 0.5 mm. (**d**) Level IV: Small foramina are visible within the depressions (arrowheads). (**e**) Level V: Narrow intraosseous canals continue from these foramina (arrowheads).

CT/CBCT demonstrated a bilateral, asymmetric osseous correlate confined to the superior medial segment of the marked course. Figure 3 shows its three-dimensional surface configuration and bony-plane angulation; Figure 4 shows the corresponding axial morphology and measurements.

Measurements are approximate descriptive values and should be interpreted in the context of image resolution, reconstruction, and section orientation. These axial sections are the cross-sectional expression of the surface morphology shown in Figure 3.

No continuous bony ridge followed the entire marked surface course. The superior osseous correlate therefore did not explain the full oblique continuation toward the middle-lower infraorbital–maxillary region.

Soft-tissue CT windows showed an oblique soft-tissue structure along part of the marked course. Its orientation corresponded more closely to the middle-lower clinical line than did the superior osseous relief.

### 3.4. Line-Guided Ultrasound: Superficial OOc-Related Muscular Component

Line-guided B-mode ultrasound showed a superficial band-like structure coursing along the medial and inferior border of OOc. Its echogenicity was comparable with adjacent mimetic muscles, and its internal architecture showed an organized directional fibrillar pattern rather than homogeneous echotexture. The structure was reproducibly visualized in long- and short-axis orientations and remained continuous within the examined segments. These features supported interpretation as an OOc-related muscular component.

**Figure 5:**
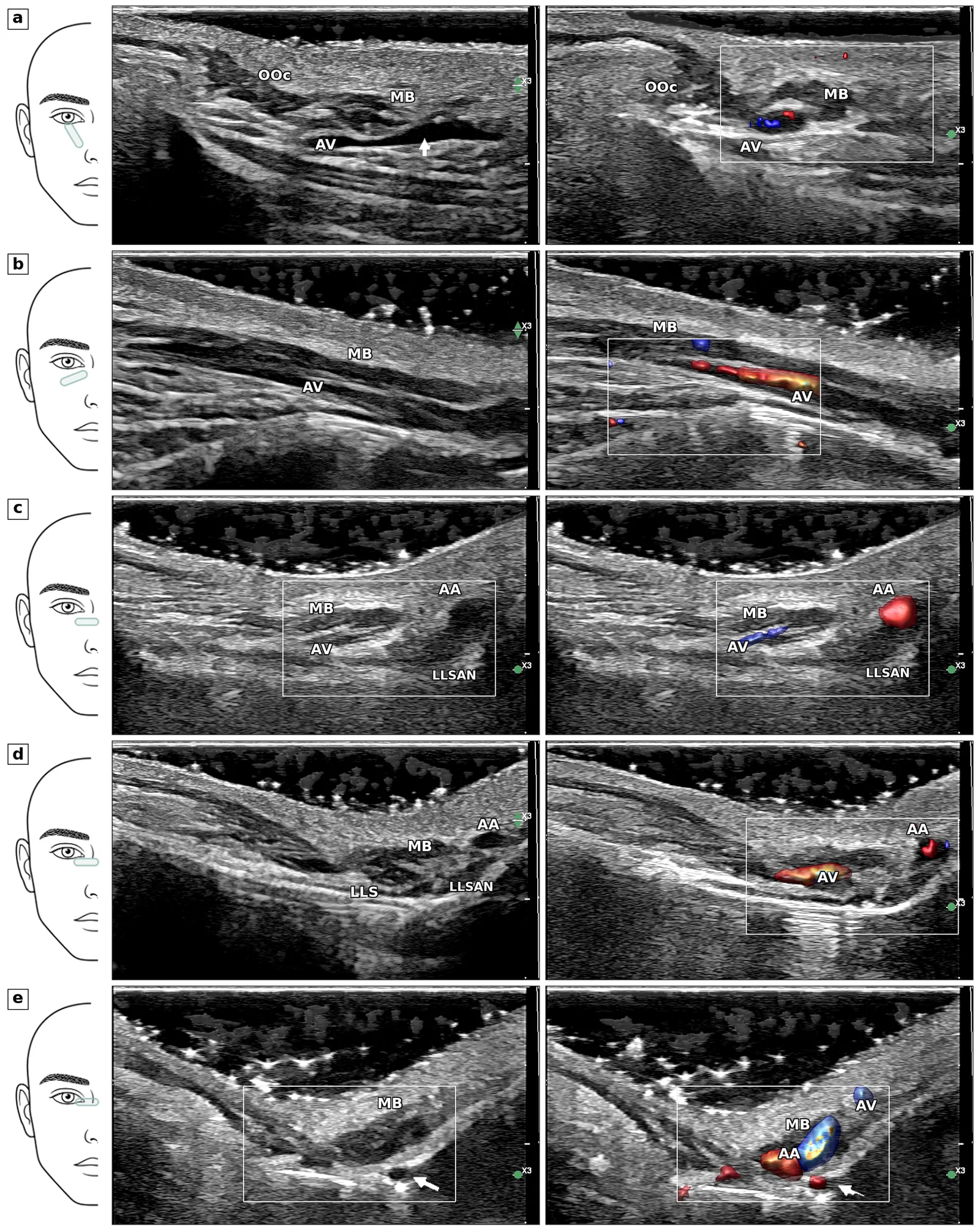
Line-guided high-frequency ultrasound and qualitative color Doppler mapping of the orbicularis oculi (OOc)-related band-like correlate and angular vessels. Ultrasound was performed relative to the clinically marked contour; all panels show the left side. Labels identify the OOc-related band-like correlate, designated MB (medial orbicularis oculi–related band), angular vein (AV), angular artery (AA), and the adjacent levator labii superioris alaeque nasi/levator labii superioris (LLSAN/LLS) field. Probe orientation follows the reference system of Kim et al. [12]; detailed point and line definitions are provided in the Supplementary Technical Note. Where visible, MB lies superficial to the LLSAN/LLS field with an intervening adipose plane. (**a**) Transverse section near the midpupillary-line reference (PL2). The arrow indicates a superficial venous tributary passing beneath MB; this relationship is further shown in Supplementary Figure S1 and correlated on CT in Figure 6. (**b**) Longitudinal section along MB between P6 and P7; AV lies deeper. (**c**–**e**) Consecutive transverse views proceeding superiorly toward the frontal process of the maxilla and medial tear trough. Arrows indicate bony canal openings and emerging vessels associated with AA; in panel (**e**), the arrow indicates a bony canal and a vessel emerging from its opening. Ultrasound showed more small bony foramina/canal openings along the examined course than were apparent on the corresponding CT images.

Within the examined course, the component corresponded to the visible and palpable line and lay superficial to the LLSAN/LLS field, separated from these muscles by an intervening adipose plane (Figure 5). Across ultrasound and soft-tissue CT correlation, the component was more superficial and more clearly delineated on the left, corresponding to the side with greater clinical conspicuity.

**Figure 6:**
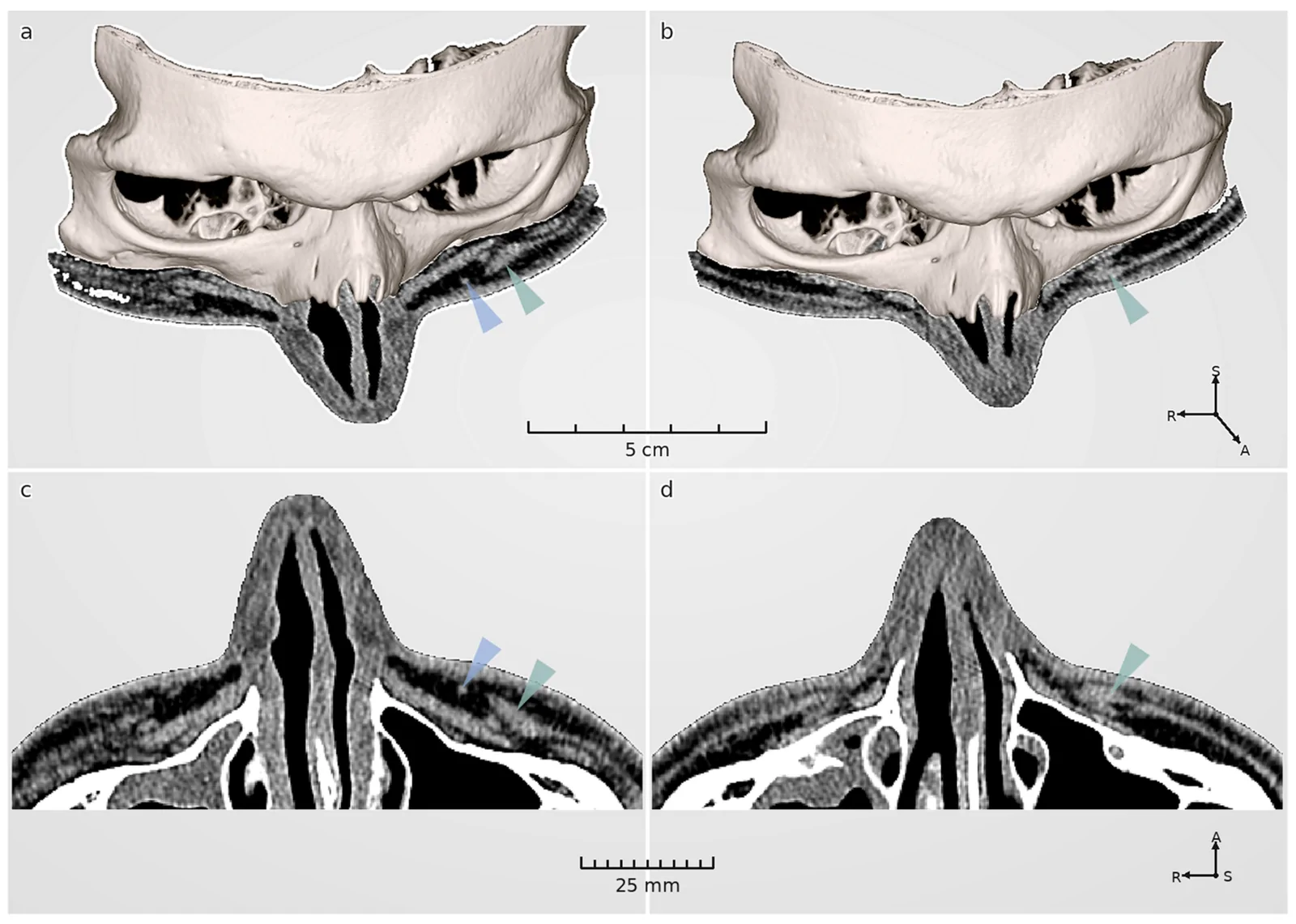
Soft-tissue CT correlation of the OOc-related band-like correlate and superficial venous pathways. Axial CT sections are shown with soft-tissue window settings. Green arrows indicate the soft-tissue correlate corresponding to MB; blue arrows indicate a superficial venous tributary or superficial vein. (**a**,**c**) Approximately 2 mm superior to the upper margin of the infraorbital foramen, the soft-tissue correlate corresponding to MB is visible with a superficial venous tributary beneath it, connecting with the deeper angular venous pathway. (**b**,**d**) Inferiorly, the oblique soft-tissue correlate continues while superficial vessels run in subcutaneous fat and drain into the angular vein (AV). Identification of MB in these panels is based on cross-modal correlation; CT alone does not establish tissue type.

### 3.5. Qualitative Color Doppler: Vascular Relationship

Qualitative color Doppler localized a venous pathway topographically consistent with the angular venous route in a plane deeper than the band-like correlate. The vascular signal remained deeper and did not coincide with the visible surface line.

At approximately the midpupillary reference level, ultrasound also identified a superficial venous tributary that passed beneath the medial orbicularis oculi–related band (MB) and joined the angular venous pathway; corresponding superficial vessels draining into the angular vein were visible on soft-tissue CT (Figure 5 and Figure 6; Supplementary Figure S1).

### 3.6. Integrated Topographic Correlation

Taken together, the findings showed a clinically continuous course with segmental, plane-specific correlates. The narrower line persisted despite a reduction in diffuse fullness and was visible in earlier photographs. Superiorly, nasomaxillary osseous microrelief coexisted with the OOc-related band-like correlate. More inferiorly, the band-like correlate provided the principal spatial correspondence; it lay superficial to the LLSAN/LLS field with an intervening adipose plane, while the angular venous pathway remained deeper and a superficial tributary passed beneath the band to join it.

The left side combined greater clinical conspicuity, a more superficial and more clearly delineated band-like correlate, and a smaller clinical response after symmetrical BoNT. The osseous findings were bilateral and asymmetric but confined to the superior segment. This cross-modal concordance supported a soft-tissue contribution to side-to-side surface expression without establishing a single causal substrate. Clinical continuity therefore did not correspond to continuity of one anatomical structure.

## 4. Discussion

### 4.1. From Procedural Attribution to Anatomical Classification

The temporal relationship to treatment made a procedural explanation reasonable, but the accumulated evidence limited its range. Diffuse fullness decreased after hyaluronidase while the narrower line retained its geometry; retrospective photographs showed similar earlier topography; and the anatomical correlate changed along the course. Together, these observations shifted the question from which procedure had created the contour to which layers made it clinically readable.

The distinction is not between a procedural and an anatomical explanation as mutually exclusive alternatives. Treatment-related changes in volume and tissue conditions may have increased the visibility of a pre-existing boundary. In this patient, the clinically new event may therefore have been recognition and classification of the contour rather than formation of a new structure.

### 4.2. The Superior Osseous Field and the Mechanobiological Question

The superior osseous correlate lies within a nasomaxillary region known for variable microrelief. Macalister described a “vascular line”, bony fissures, and small vascular foramina in this field [14], while later work by Rusu et al. placed sutura notha, Macalister’s foramina, and related small canals within the same regional spectrum [15,16]. These descriptions provide a morphological reference for the observed field rather than a patient-specific diagnosis.

Local bony-plane angulation may be as important as the depth of an individual depression. The more pronounced angulation on the right persisted farther inferiorly, whereas the left-sided depression widened, flattened, and faded. Such differences can alter support geometry beneath thin, poorly buffered soft tissue. Because the osseous correlate was confined to the superior segment, however, bone could contribute only to the upper part of the clinically continuous contour.

A further, explicitly mechanobiological question arises from the local coexistence of osseous microrelief and the superficial OOc-related band. General enthesis literature shows that recurrent loading at soft-tissue attachments can influence bony-surface organization [17,18]. By analogy, repeated tension through this region could influence the expression or organization of pre-existing microrelief and the overlying soft tissues, but this remains a testable hypothesis rather than evidence of patient-specific remodeling. Supplementary Figure S2 shows a more strongly expressed osteological analogue of the gutter-like depression and bony-plane angulation.

### 4.3. The Superficial OOc-Related Muscular Component: Support, Contour, and Volume-Dependent Visibility

The course and layered position of the observed component place it closest to the medial muscular band of OOc described by Park et al., whose variants followed the medial tear-trough edge and descended toward the cheek along the nasojugal axis [19]. Park provides the closest topographic comparator. Broader functional interpretation comes from the newer OOc support-system literature [9] and descriptions of the malaris muscle and related midface support [20,21,22]. Together, these sources support viewing comparable medial fascicles as part of the dynamic architecture of the lower eyelid–cheek transition. Such a component could contribute to OOc position, suborbicularis oculi fat (SOOF) tension, resistance to descent, and midface convexity. Its superficial, tension-bearing course could also become visible through a thin tissue envelope as a linear contour.

Limited soft-tissue buffering may allow even a small support element to sharpen contrast with the adjacent hollow and optically deepen the tear trough without true volume loss. The component need not form the entire trough to modify its readability. If its course is relatively stable, variation in SOOF and deep medial cheek support, malar convexity, tissue hydration, or added volume could buffer or accentuate the edge without changing its topography [3,4,5,20,21,22].

### 4.4. Muscle–Vein Topography and the Hypothesis of a Limited Perivenous Space

Calomeni et al. provide functional context for the observed muscle–vein relationship, showing in vivo that the angular vein may course within OOc and that its caliber and flow can change during facial expression [3]. Iwanaga et al. described an alternative angular-vein valve model in which OOc, depressor supercilii (DS), and zygomaticus minor may form sites of dynamic narrowing along the venous route [11]. These studies establish regional plausibility rather than the mechanism in this patient.

**Figure 7:**
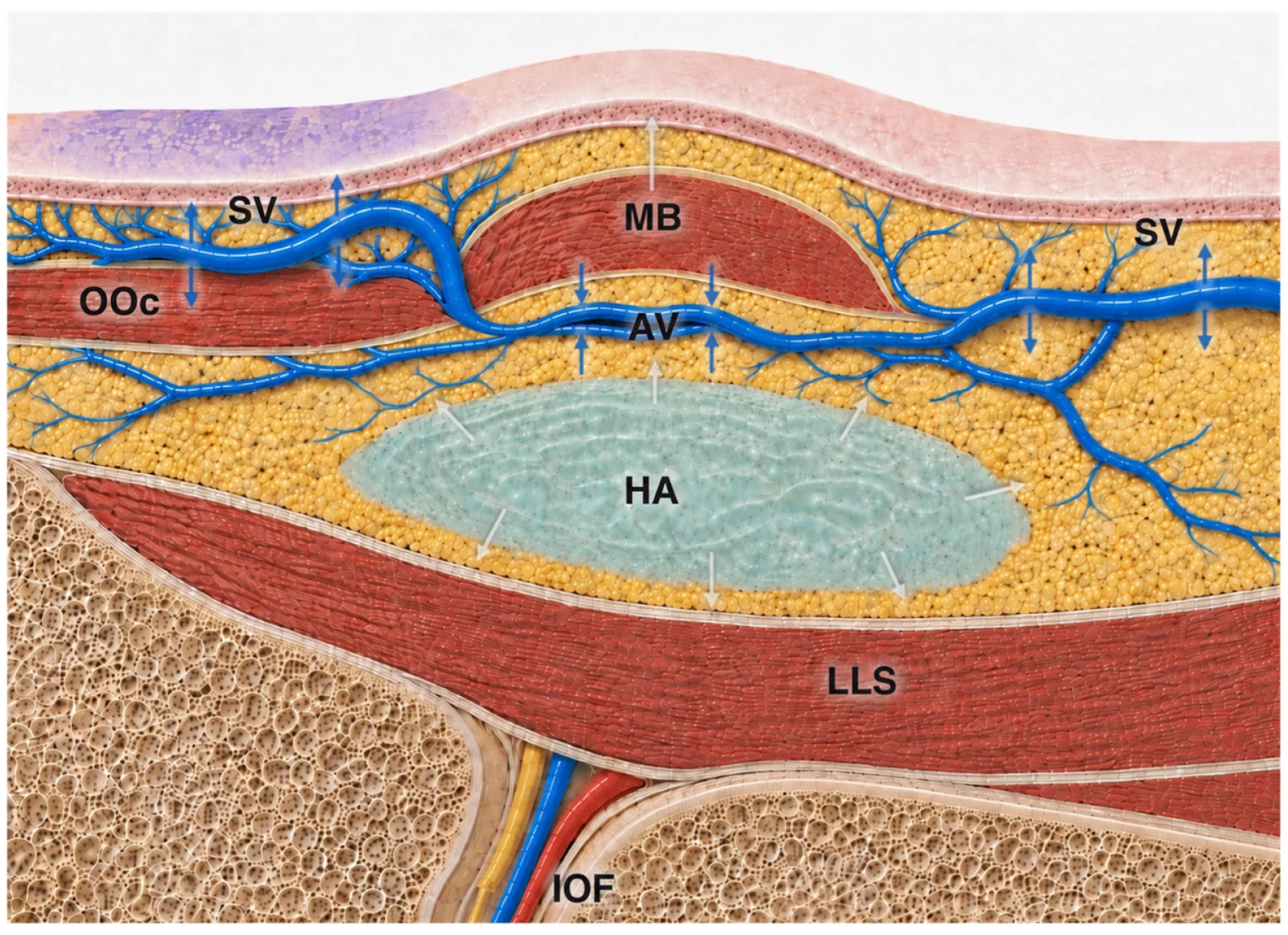
Conceptual model of the limited perivenous-space hypothesis. At the tissue scale, deep volume expansion, hydration, or edema may reduce local soft-tissue buffering reserve, increase superficial displacement or tension of the medial orbicularis oculi–related band (MB), sharpen the edge, and increase edge–hollow contrast without requiring additional volume loss. At the venous scale, the superficial band overlies the angular venous route and local tributaries; reduced perivenous spatial reserve may alter local compliance and filling, increasing the clinical visibility of superficial tributaries and small cutaneous branches. White arrows indicate tissue or MB displacement/tension; blue arrows indicate venous-pathway consequences. Abbreviations: AV, angular vein; HA, hyaluronic acid; IOF, infraorbital foramen; LLS, levator labii superioris; MB, medial orbicularis oculi–related band; OOc, orbicularis oculi; SV, superficial venous tributary.

In this patient, the band-like component formed a local superficial cover over the deeper venous route from the medial canthus toward the pupillary level, while a superficial tributary passed beneath it and joined the angular pathway. This configuration provides the anatomical basis for a limited perivenous-space hypothesis (Figure 7).

In that model, HA-related hydration, edema, or increased tissue volume beneath the superficial cover could reduce spatial reserve around the vein and its tributary, lowering local compliance and buffering capacity. The superficial band may organize the geometry of the surface edge, whereas changes in venous and perivenous filling could modify the visibility and optical quality of the same corridor.

### 4.5. Dynamic Expression, BoNT, and the Shield Hypothesis

The described OOc–LLSAN connections and DS–LLSAN–OOc continuity provide a possible route for regional tension transfer [23,24]. The oblique course of LLSAN and the superior segment of the medial OOc band, together with convergence of their likely tension vectors, also provides an anatomical rationale for participation in the oblique component of bunny lines [23,24,25]. Through this connected system, DS contraction and LLSAN activity may increase tension in medial OOc fibers and the band, increasing its surface readability. The band need not move for its visibility to change; altered network tension may be sufficient. BoNT can therefore function as a dynamic probe of this relationship.

On the side where the muscular component was more superficial, the response after symmetrical BoNT was weaker: the nasolabial fold flattened less, the oral commissure remained relatively higher, and the same direction of asymmetry was observed again at later reassessment. These findings were consistent with a smaller functional change in the wider LLSAN/LLS–medial midface system. The shield hypothesis proposes that a superficial OOc-related component may act as a local topographic cover, limiting uniform access, diffusion, or distribution of toxin to the deeper LLSAN and LLS and thereby favoring an incomplete or asymmetric response. This is a topographic delivery hypothesis rather than a demonstrated diffusion barrier.

**Figure 8:**
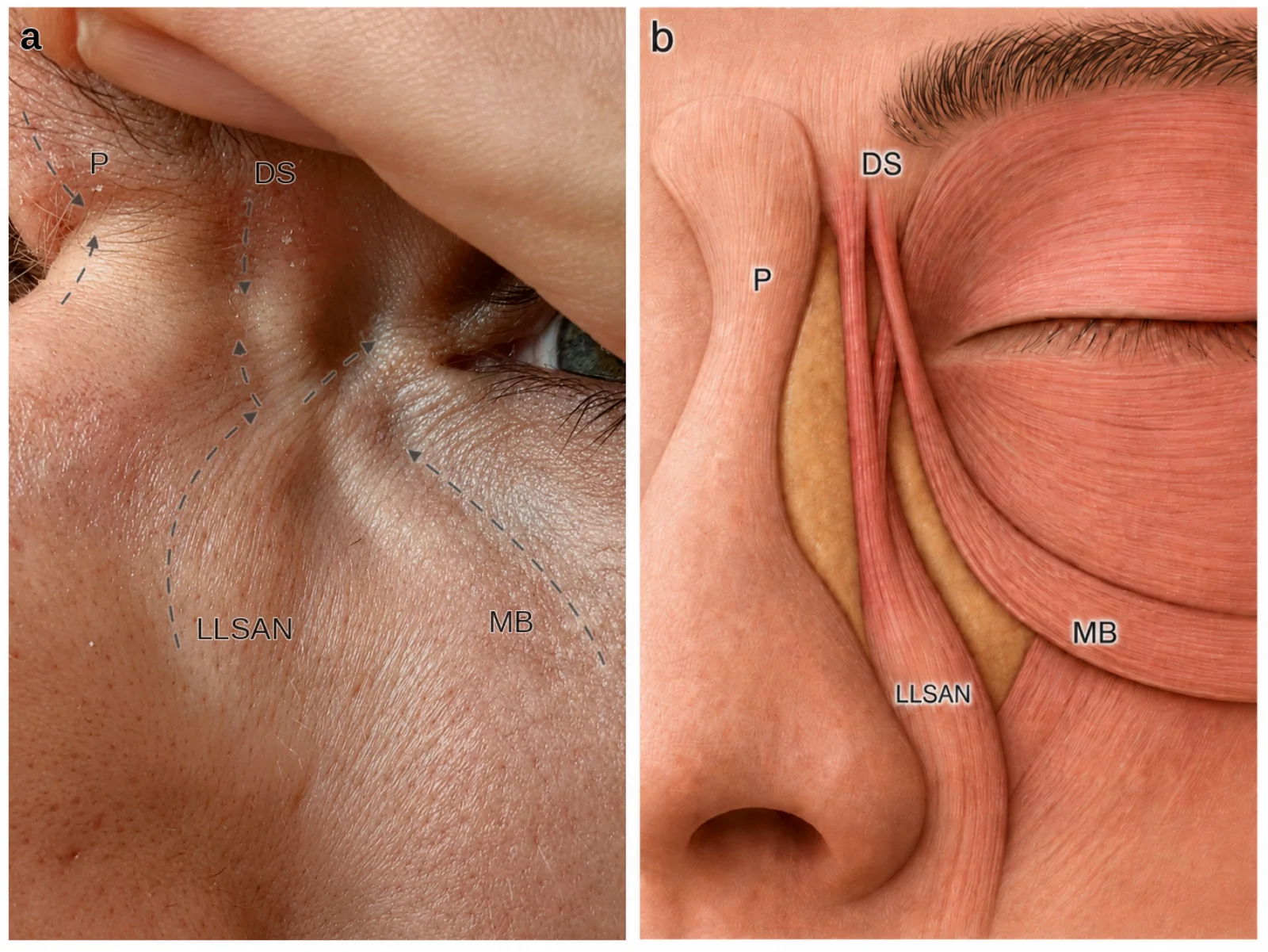
Dynamic recruitment of the medial infraorbital contour and its interpretation in relation to depressor supercilii–levator labii superioris alaeque nasi–orbicularis oculi (DS–LLSAN–OOc) continuity. (**a**) Patient-specific dynamic morphology during recruitment of the medial mimetic field. The medial end of the eyebrow was manually elevated, followed by maximal glabellar/frowning contraction and elevation of the nasal ala and upper lip. Labels identify the procerus (P), depressor supercilii (DS), levator labii superioris alaeque nasi (LLSAN), and the medial orbicularis oculi–related band (MB). Arrows indicate muscle-tension and traction vectors interpreted from the maneuver, including downward traction of the medial brow and superomedial traction of the medial infraorbital and upper lateral nasal skin. Contraction-related muscular relief is visible through the thin superficial tissues. (**b**) Anatomical schematic based on the DS–LLSAN–OOc connections described by Hur et al. [23,24], used as an interpretive model for the muscular configuration and dynamic recruitment pattern observed in this patient. The scheme illustrates an anatomically plausible route of tension transfer within this field but does not establish that the patient had the exact anatomical variant depicted.

The possible aesthetic effect is bidirectional. Reducing unfavorable band tension could soften the oblique bunny-line component, reduce band conspicuity, and lessen edge–hollow contrast. Conversely, if the same band contributes to medial OOc position and OOc–SOOF support [9,19,20,21,22], excessive relaxation could reduce support, alter midface projection, or lengthen the lower eyelid–cheek transition. The band is therefore a plausible but non-predictive modifier rather than a self-evident treatment target. Figure 8 juxtaposes the patient-specific pattern of dynamic recruitment and muscle-tension vectors with the DS–LLSAN–OOc connections described by Hur et al. as an anatomical interpretive model.

### 4.6. Classification Before Correction

Before correction is considered, two questions should be separated: what organizes the geometry of the contour, and what increases its current visibility. This distinction follows from the observed dissociation between the relatively stable course of the contour and its variable clinical readability. The first question concerns the anatomical element or interplanar relationship that gives the contour its course; the second concerns changes in tissue volume and hydration, edema, and mimetic tension that modulate its clinical conspicuity.

A similar surface appearance may lead to opposite clinical decisions depending on the layer that organizes the contour and the factor that increases its visibility. An intervention directed at such a modifier may leave the contour geometry unchanged, whereas modification of a supporting element may also alter relationships between adjacent planes. Mapping therefore serves classification rather than automatically selecting treatment.

## 5. Limitations

The single-patient design does not establish prevalence, the range of anatomical variation, or reproducibility across patients. Its contribution is the mapped relationship among clinical topography, cross-sectional imaging, ultrasound, and vascular localization in one in vivo configuration.

Using the same marked surface course across modalities improved spatial registration but also introduced anchoring risk. Ultrasound provided sonographic rather than histological characterization, while CT offered limited soft-tissue discrimination. Small CT/CBCT features and submillimetric measurements remain constrained by spatial resolution, reconstruction, section orientation, and partial-volume effects. Dedicated high-resolution magnetic resonance imaging could have added another soft-tissue modality but would not by itself establish histological identity or assignment to a specific anatomical variant.

Color Doppler was used qualitatively for topographic localization rather than quantitative hemodynamic assessment. Retrospective photographs and the BoNT comparison were not acquired under a standardized prospective protocol and support qualitative observations rather than causal treatment inference. Persistence of the narrower contour after hyaluronidase distinguished it clinically from the diffuse fullness, although complete HA dissolution was not independently verified.

The proposed periosteal–fascicular coupling, edge–hollow effect, limited perivenous-space mechanism, and shield interpretation remain hypotheses. Larger imaging series, standardized dynamic assessment, higher-resolution soft-tissue imaging, and anatomical or histological correlation are needed to determine their reproducibility and range.

## 6. Conclusions

In this patient, a clinically continuous medial infraorbital–maxillary contour corresponded to segmental, plane-specific findings: a limited superior nasomaxillary osseous correlate, a superficial OOc-related band-like correlate along the medial and inferior border of OOc, and a deeper angular venous pathway with a superficial tributary crossing beneath the band. Their changing contribution along the course supports interpretation as a layered boundary rather than a single-substrate line.

Changes in surrounding volume may have amplified the visibility of a pre-existing configuration without creating its full anatomical course. The OOc-related component may have modified tear-trough visibility by sharpening a surface edge adjacent to the hollow. In similar clinical situations, identifying the contributing layer and the factor that modifies its visibility may be useful before corrective treatment is considered.

## Data Availability

The data supporting the findings of this study are not publicly available because they include patient imaging, clinical photographs, and medical information. Deidentified data may be made available from the corresponding author upon reasonable request, subject to privacy, ethical, and legal restrictions.

## Supplementary Materials

The following supporting information can be downloaded at: https://www.mdpi.com/article/10.3390/jcm15166268/s1. Supplementary Technical Note: CT/CBCT Acquisition, Reconstruction, and Measurement; Ultrasound Topographic Orientation, Sonographic Measurements, and Image-Specific B-Mode and Color Doppler Settings. Supplementary Figure S1. Ultrasound documentation of a superficial venous tributary and its relationship to the orbicularis oculi (OOc)-related band-like correlate, designated MB (medial orbicularis oculi–related band), and the angular vein. (a) Transverse section showing a superficial venous tributary (SV) passing beneath MB, with the angular vein (AV) located deeper. (b) Longitudinal section showing the course of SV and its confluence with AV. Supplementary Figure S2. Osteological analogue of nasomaxillary gutter-like microrelief and bony-plane angulation. An arrowhead indicates the gutter-like depression. The specimen illustrates a more strongly expressed configuration of the depression and adjacent raised anterior bony boundary, together with angulation between the lateral surface of the frontal process of the maxilla and the plane of the medial orbital rim. The specimen is from the Human Anatomy Collection of the Institute of Human Anatomy and Histology, Department of Biomedicine, Neuroscience and Advanced Diagnostics (Bi.N.D.), University of Palermo. Published under permission.

## Abbreviations

AA, angular artery; AV, angular vein; BoNT, botulinum neurotoxin; CBCT, cone-beam computed tomography; CT, computed tomography; DS, depressor supercilii; FPM, frontal process of the maxilla; HA, hyaluronic acid; IOF, infraorbital foramen; LLS, levator labii superioris; LLSAN, levator labii superioris alaeque nasi; MB, medial orbicularis oculi–related band; OOc, orbicularis oculi; P, procerus; PL2, perpendicular reference line through the midpupil; SOOF, suborbicularis oculi fat; SV, superficial venous tributary.
