# Supplementary material for: An Orbicularis Oculi-Related Band as a Potential Modifier of Tear-Trough Visibility: A Hypothesis-Generating Observation of a Layered Boundary in a Patient
Source: J Clin Med. 2026 Aug 13;15(16):6268. doi: 10.3390/jcm15166268 (PMC13513525; doi:10.3390/jcm15166268)
Supplement: Supplementary file 1 [file jcm-15-06268-s001.zip › jcm-4414536-supplementary.pdf]

Supplementary Technical Note: CT/CBCT Acquisition, Reconstruction, and Measurement; Ultrasound Topographic Orientation, Sonographic Measurements, and Image-Specific B-Mode and Color Doppler Settings

1. CBCT acquisition and reconstruction

CBCT of the maxillofacial region was acquired on a RAYSCAN Studio system (RAY Co., Ltd., Hwaseong-si, Gyeonggi-do, Republic of Korea) using the Facial protocol. Acquisition settings were 90 kVp, 9.0 mA, and 16 s. Images were reconstructed with a 0.30-mm voxel size. The reconstructed field of view, estimated from the Digital Imaging and Communications in Medicine (DICOM) matrix and pixel spacing, was approximately 20 × 20 cm. DICOM datasets were reviewed in Xelis Dental 1.0.6.3 and 3D Slicer 5.10.0 using multiplanar and three-dimensional reconstructions.

2. CT acquisition and reconstruction

CT was acquired on an Optima CT660 scanner (GE Medical Systems, LLC, Waukesha, WI, USA) in helical mode using the local sinus CT protocol. Acquisition settings were 120 kVp, 90 mA, 0.6-s rotation time, pitch factor 0.531, 0.625-mm single collimation, 20-mm total collimation width, and 0° gantry tilt. The high-resolution axial "0.625 mm Bone" series was reconstructed with the BONEPLUS bone kernel at 0.625-mm slice thickness, 0.312-mm reconstruction interval, 207-mm reconstruction diameter, a 512 × 512 matrix, and 0.404 × 0.404-mm pixel spacing. The DICOM dataset was reviewed in 3D Slicer 5.10.0 using multiplanar and three-dimensional reconstructions.

3. CT volume orientation, bone segmentation, and three-dimensional reconstruction

The CT volume was anatomically reoriented before analysis. Bone segmentation was performed in 3D Slicer 5.10.0 using attenuation thresholds adjusted to osseous structures. The segmentation was then refined manually with selection and editing tools to preserve fine, sharply defined surface features that thresholding alone could omit. The three-dimensional reconstruction was used for spatial orientation, morphologic assessment, and preliminary selection of observation levels; final measurements were not obtained from the reconstructed surface.

4. Selection of CT observation levels and axial measurements

The levels shown in Figures 3 and 4 were selected where individual elements of the local osseous microrelief were most clearly delineated or showed their greatest local expression. In Figure 3, reference levels I–V correspond sequentially to panels (a–e) in Figure 4. Candidate sites were identified on the three-dimensional reconstruction and then verified and, where necessary, corrected on the corresponding axial CT sections. Final measurement points were placed on the axial images at the sharpest and most locally prominent depression or ledge profiles. Measurements were obtained with the Markups Line tool in 3D Slicer. The reported values

represent maximum local dimensions of the best-demarcated features rather than regional averages.

5. Ultrasound system and transducer

High-frequency ultrasound was performed on a Philips EPIQ Elite system (Philips Ultrasound LLC, Bothell, WA, USA) with an mL26-8 compact linear broadband transducer using the Dermal preset for superficial imaging. The transducer has a nominal bandwidth of 26–8 MHz. B-mode ultrasound and qualitative color Doppler imaging were used for line-guided correlation with the clinically marked contour.

6. Topographic orientation according to Kim et al.

Topographic orientation followed the facial mapping system of Kim et al. [12]. PL1 is the perpendicular reference line through the medial canthus, PL2 the perpendicular reference line through the midpupil, and TL3 the transverse reference line through the inferior orbital rim. P6 is the intersection of TL3 with PL1, and P7 the intersection of TL3 with PL2. These labels were used to report probe position and orientation. Cross-modal correlation remained anchored to the clinically marked contour, with the probe aligned along its long and short axes.

7. Sonographic course and depth of the OOc-related band-like correlate

At PL1, the OOc-related band-like correlate was located 0.23 cm beneath the skin. It followed the clinically marked contour obliquely inferolaterally toward PL2, where its depth increased to 0.38 cm. Lateral to PL2, the correlate coursed progressively deeper and its fascicular echotexture became less distinct.

8. Venous pathway and superficial tributary

In the PL1–PL2 segment, the dominant longitudinal venous signal followed the direction of the band in a deeper plane and was interpreted as the angular vein on the basis of its course and layer. A second superficial channel coursed approximately vertically in the midpupillary projection at a depth of about 0.4 cm. Near the level of the infraorbital foramen, it turned deeper, passed beneath the band, and joined the angular venous pathway. Continuity with the angular venous pathway supported its interpretation as a superficial venous tributary.

9. B-mode settings recorded across displayed panels

The dedicated B-mode tiles in Figure 5 panels (a), (b), and (d) were acquired with the Dermal preset and mL26-8 transducer. Interface-specific setting labels are reproduced as displayed on the archived ultrasound images. The displayed settings were frame rate 32 Hz, 2D gain 54%, dynamic range R60, persistence/resolution P Low Res, RS, AS 0/0, thermal index for soft tissue (TIS) 0.1, mechanical index (MI) 0.6, grayscale map M3, and ×3 magnification.

10. Color Doppler display settings recorded across displayed panels

The displayed color Doppler tiles used a bidirectional color-flow map and were acquired with the Dermal preset and mL26-8 transducer. Settings common to all color Doppler tiles were frame rate 9 Hz, 2D gain 55%, dynamic range R55, persistence/resolution P Med Res, AS 0/0, pulse-repetition frequency 1039 Hz, wall filter 52 Hz, displayed Doppler frequency 10.0 MHz,

flow-velocity setting FV Med, a symmetric velocity scale of −4.0 to +4.0 cm/s with the baseline centered at zero, MI 0.8, display maps M3/M3, and ×3 magnification. Color-flow gain (CF) was 59% and TIS was 0.5 in the right tile of Figure 5 panel (a); CF was 43% and TIS was 0.4 in the right tiles of panels (b) and (d) and in both tiles of panels (c) and (e). Color Doppler mode was active in the left tiles of panels (c) and (e), although no color signal was visible in those frames.

11. Technical scope

The color Doppler assessment was qualitative and topographic; no quantitative hemodynamic measurements were performed.

Supplementary Figure Legends

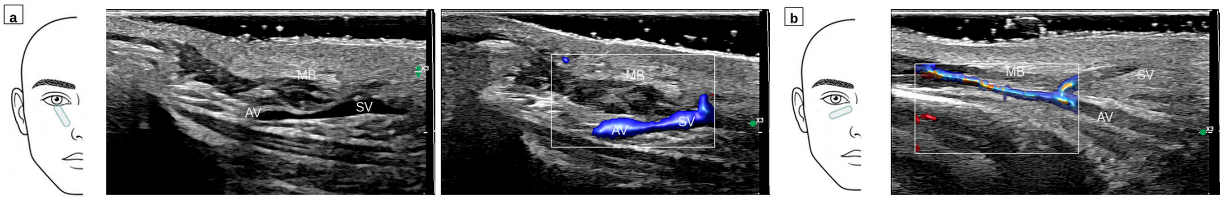

Supplementary Figure S1. Ultrasound documentation of a superficial venous tributary and its relationship to the orbicularis oculi (OOc)-related band-like correlate, designated MB (medial orbicularis oculi–related band), and the angular vein. (a) Transverse section showing a superficial venous tributary (SV) passing beneath MB, with the angular vein (AV) located deeper. (b) Longitudinal section showing the course of SV and its confluence with AV.

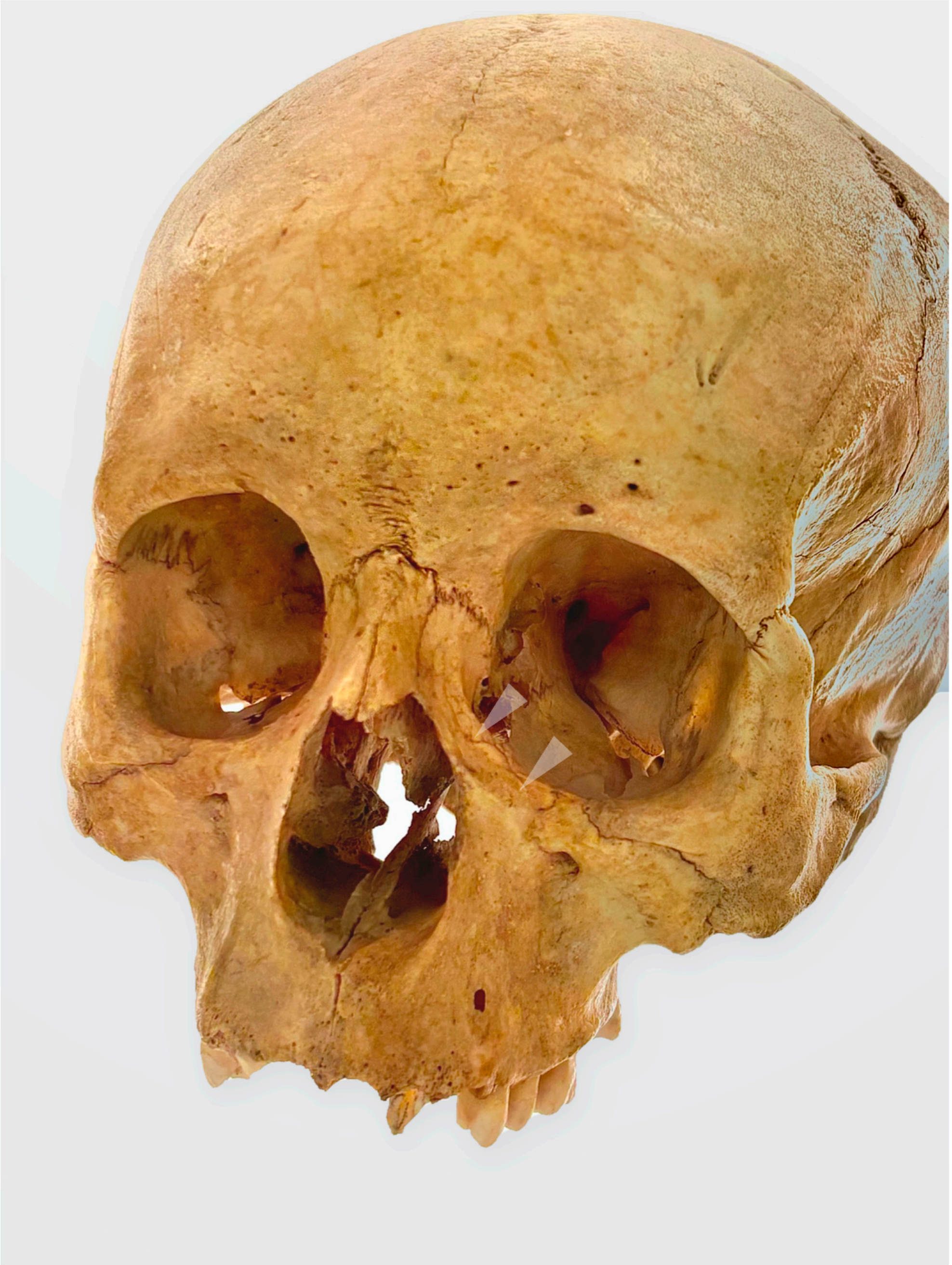

Supplementary Figure S2. Osteological analogue of nasomaxillary gutter-like microrelief and bony-plane angulation. An arrowhead indicates the gutter-like depression. The specimen illustrates a more strongly expressed configuration of the depression and adjacent raised anterior bony boundary, together with angulation between the lateral surface of the frontal process of the maxilla and the plane of the medial orbital rim. The specimen is from the Human Anatomy Collection of the Institute of Human Anatomy and Histology, Department of Biomedicine, Neuroscience and Advanced Diagnostics (Bi.N.D.), University of Palermo. Published under permission.
